# Detection of nitrogen deficiency QTL in juvenile wild barley introgression lines growing in a hydroponic system

**DOI:** 10.1186/1471-2156-13-88

**Published:** 2012-10-20

**Authors:** Astrid Hoffmann, Andreas Maurer, Klaus Pillen

**Affiliations:** 1 Institute of Agricultural and Nutritional Sciences, Chair of Plant Breeding, Martin-Luther-University Halle-Wittenberg, Betty-Heimann-Str. 3, Halle, 06120, Germany

**Keywords:** Introgression lines, *Hordeum vulgare* ssp. *spontaneum*, QTL, Nitrogen stress tolerance, Hydroponics, Juvenile plant development

## Abstract

**Background:**

In this report we studied the genetic regulation of juvenile development of wild barley introgression lines (S42ILs) under two contrasting hydroponic nitrogen (N) supplies. Ten shoot and root related traits were examined among 42 S42ILs and the recurrent parent ‘Scarlett’. The traits included tiller number, leaf number, plant height, leaf and root length, leaf to root length ratio, shoots and root dry weight, shoot to root weight ratio, and chlorophyll content. Our aims were (1) to test the suitability of a hydroponic system for early detection of favourable S42ILs, (2) to locate quantitative trait loci (QTL) that control the examined traits, (3) to identify favourable wild barley alleles that improve trait performances in regard to N treatment and, finally, (4) to validate the identified QTL through comparison with previously reported QTL originating from the same parental cross.

**Results:**

The phenotypic data were analysed in a mixed model association study to detect QTL. The post-hoc Dunnett test identified 28 S42ILs that revealed significant (*P* < 0.01) effects for at least one trait. Forty-three, 41 and 42 S42ILs revealed effects across both N treatments, under low N and under high N treatment, respectively. Due to overlapping or flanking wild barley introgressions of the S42ILs, these associations were summarised to 58 QTL. In total, 12 QTL of the hydroponic N study corresponded to QTL that were also detected in field trials with adult plants of a similar S42IL set or of the original S42 population. For instance, S42IL-135, -136 and -137, revealed increasing *Hsp* effects for tiller number, leaf number, leaf length, plant height and leaf to root ratio on the long arm of chromosome 7H. These QTL correspond to QTL for ears per plant and plant height that were previously detected in field trials conducted with the same S42ILs or with the S42 population.

**Conclusion:**

Our results suggest that the QTL we identified under hydroponic N cultivation partly correspond to QTL detected in field experiments. Due to this finding, screening of plants in early developmental stages grown in a hydroponic system may be a fast and cost effective method for early QTL detection and marker-assisted allelic selection, potentially speeding up elite barley breeding programs.

## Background

The domestication of barley, starting about 10,000 years ago [1], led to a multitude of genotypes, which are well adapted to farming under a broad range of conditions [2]. Besides, intensive barley breeding for over a century led to modern barley cultivars revealing a low degree of genetic diversity [3]. One aspect in innovating modern breeding programs is the introduction of exotic germplasm into new varieties [4]. The *mlo* powdery mildew resistance is a prominent example for the introduction of favourable wild barley alleles into elite barley varieties [5]. In this regard, Tanksley and Nelson [6] established the advanced backcross (AB) quantitative trait loci (QTL) approach in tomato, by which exotic germplasm is introduced into modern cultivars and QTL are characterised in the AB populations. A further approach to detect QTL with exotic germplasm is described by Zamir [7]: the establishment of lines that carry single chromosomal introgressions from exotic donors, so called introgression lines (ILs). In comparison to AB populations, the genetic background of ILs is fixed to the recurrent elite parent. Thus, a complete set of ILs represents the entire donor genome on the background of the recurrent parent. Compared to other mapping populations like recombinant inbred lines (RILs), ILs offer a higher statistical power and allow the detection of small QTL effects [8]. Within the last years, IL sets were developed for important crop species like maize [9], wheat [10] and rice [11].

In barley, von Korff et al. [12] crossed the German spring barley cultivar ‘Scarlett’ (*Hordeum vulgare* ssp. *vulgare*, *Hv*) with the Israeli wild barley accession ‘ISR42-8’ (*Hordeum vulgare* ssp. *spontaneum*, *Hsp*) to develop the BC_2_DH population S42. By further backcrossing of population S42 with ‘Scarlett’ and subsequent marker assisted selection, a set of wild barley introgression lines (S42IL) was established [13]. Different S42IL sets were used to locate *Hsp* QTL that regulate pathogen resistances, flowering time, agronomic performance, malting quality and drought stress tolerance [14-18]. Afterwards, the S42IL population was subjected to high-resolution (HR) genotyping with an Illumina 1536-SNP array and the threshability locus *thresh1* was located on chromosome 1H by means of high-resolution mapping with segregating offspring of the original S42IL [19].

Nitrogen (N) is one of the nutrients plants need in high quantity [20]. Therefore, it is necessary to understand plant stress responses to N deficiency including changes in growth, development and differentiation. Various QTL were found in field trials and greenhouse experiments with barley under N deficiency, e.g. in recombinant inbred lines (RILs) [21]. Also wild barley has been used to identify favourable alleles under N deficiency. In population S42 Saal et al. [22] identified 14 QTL with reference to N supply. For instance, the authors mapped a QTL on the long arm of chromosome 7H that caused an effect on adult plant height across both N treatments. Later on, Schnaithmann and Pillen [23] mapped QTL for chlorophyll content and N content in the same region of chromosome 7H within the S42IL population.

The plant response to N deficiency affects plant growth and development and is a crucial component of yield [24]. One major problem in studying the interaction between plant growth and N deficiency is the uncertainty of the soil N supply in the field [25]. An alternative approach is the examination of N deficiency under controlled conditions in hydroponic systems. Although research on plants under N deficiency has been carried out in hydroponics [25-27], little is known about the direct comparison between data from single crop plants under N stress in a hydroponic system and the crop performance of the same genotype in field trials and greenhouse experiments. For instance, Beatty et al. [28] reported that barley genotypes studied under field and hydroponic conditions showed similar growth and nitrogen used efficiency characteristics. They assumed that using less-variable growth environments is another way to gain knowledge about traits and genetic targets related to N nutrition. So far, a genome wide QTL analysis with a set of wild barley ILs, focussing on these questions, has not been carried out.

In our study, juvenile wild barley ILs were grown in a hydroponic system under two contrasting N treatments. By conducting the hydroponic experiments, we aimed to (1) test the suitability of our hydroponic system for early selection of favourable S42ILs, to (2) detect QTL in the S42IL population that contribute to the control of juvenile plant growth, to (3) find favourable wild barley alleles that improve trait performances either across both N treatments or under low N supply and, finally, to (4) compare the QTL results of our experiments on juvenile barley plants with QTL found in field trials and greenhouse experiments with adult plants of the S42IL and S42 populations and other QTL studies.

## Methods

### Plant material

**Figure 1:**
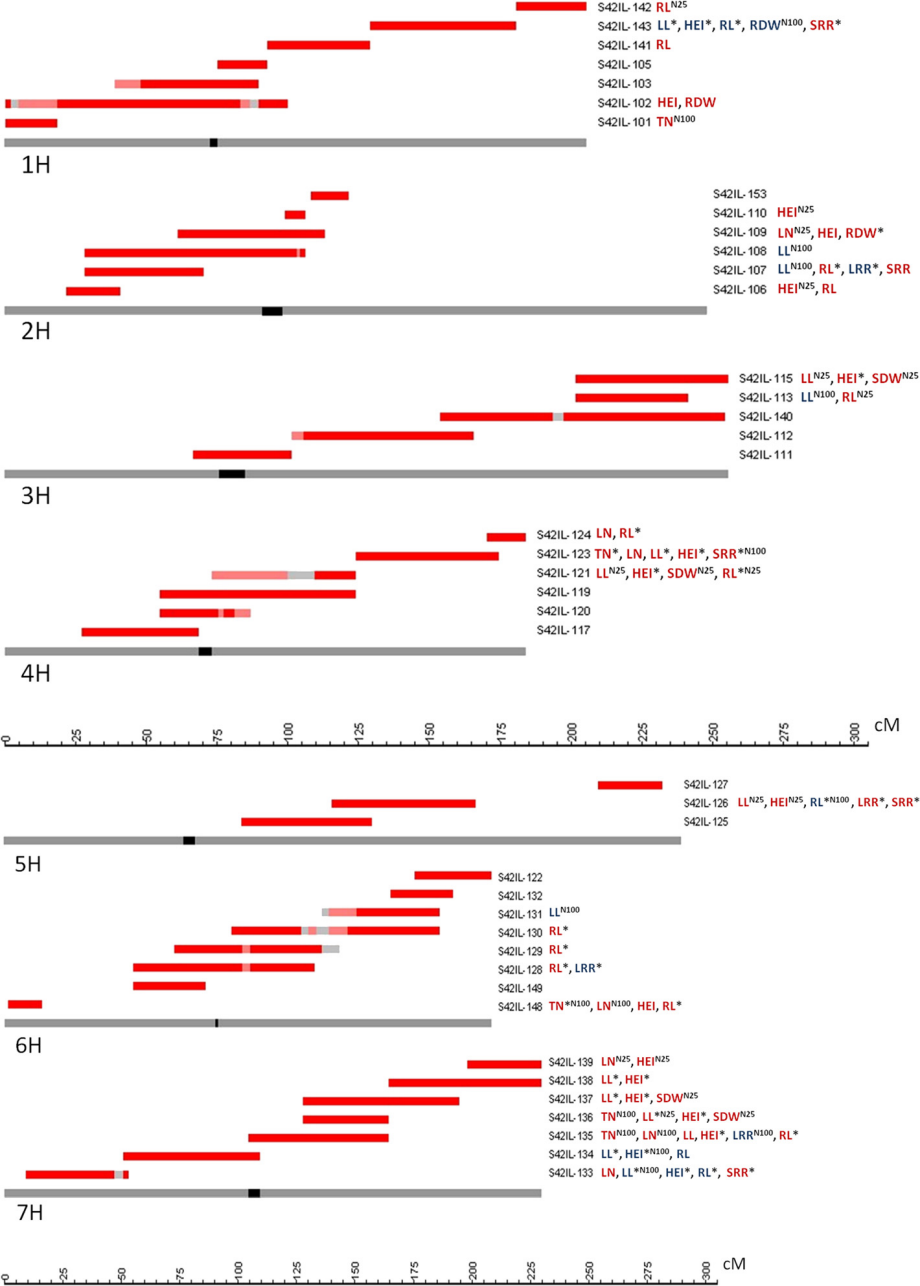
**SNP map with 58 significant (*P*** <**0**.**01) QTL effects among 42 S42ILs and nine traits.** The chromosomes 1H to 7H are indicated as grey bars. The centromeric region is indicated as a black box within the chromosome. The cM positions of the SNP loci are indicated with the ruler at the bottom, according to Schmalenbach et al. [19]. The extent of the target *Hsp* introgression of each S42IL is given above the respective chromosome. Homozygous *Hsp* loci, heterozygous *Hsp* loci and loci without SNP data are presented in red, pink and light grey, respectively. The associated QTL effects are indicated right to the introgressions with abbreviations of the nine traits, i.e. TN, LN, LL, HEI, SDW, RL, RDW, LRR, SRR. Trait abbreviations are explained in Table 2. The colour of the abbreviations indicates an increasing (red) or decreasing (blue) *Hsp* effect. Highly significant (*P* < 0.001) associations are highlighted with an asterisk. Trait associations that were only detected under one treatment are labelled with ^**N100**^ or ^**N25**^, respectively.

Our investigations on juvenile barley plants in a hydroponic system were carried out with introgression lines (ILs) of the population S42IL. The S42ILs are derived from a cross between the German spring barley (*Hv*) cultivar ‘Scarlett’ and the Israeli wild barley (*Hsp*) accession ‘ISR42-8’. Advanced backcrossing with ‘Scarlett’ and marker assisted selection with SSRs resulted in the development of a set of 59 S42ILs in BC_3_S_4_ generation as described in Schmalenbach et al. [13]. Based on genotypic data with 636 informative SNPs [19] we selected 42 representative S42ILs with target introgressions located on chromosomes 1H to 7H (Figure 1). The recurrent parent ‘Scarlett’ served as the control genotype in the hydroponic experiments conducted.

### Hydroponic plant cultivation and phenotyping

**Figure 2:**
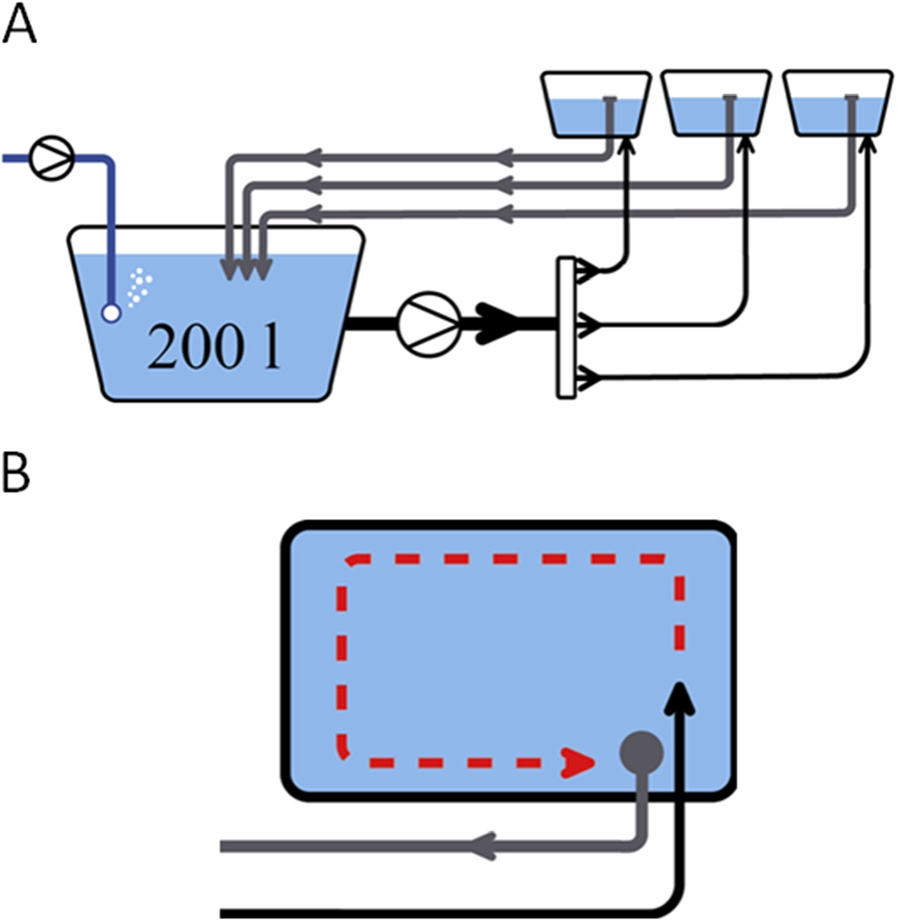
**Hydroponic system. A. ** The utilised hydroponic system included an accumulation tank for 200 l of nutrient solution that was connected to three boxes for plant cultivation. **B**. The nutrient media circulated permanently between the boxes and the tank, ensuring that within and between boxes all plants were exposed to the same nutrient composition.

Juvenile barley plants were tested during two years in six hydroponic experiments at two locations of the University of Halle. In 2008, one and two experiments were carried out under low and high N supply, respectively, in a glasshouse at the experimental station ‘Kühnfeld’, Halle. In 2011, the same experimental set up was repeated in a second glasshouse at the experimental station ‘Heide’, Halle. In each of the six experiments, an accumulation tank for the nutrient solution was connected to three boxes for plant cultivation (Figure 2). On each of the three boxes, a perforated tray was placed containing 140 holes to hold the single test plants. The outer perimeter of the tray was filled with 44 non-measured ‘Scarlett’ plants in order to reduce border effects in the box due to inhomogeneous light and space availability. The remaining 96 holes were split into two blocks of 48 holes. ‘Scarlett’, the spring barley cultivar ‘Barke’ and the 42 S42ILs grew in four, two and one replications per block, respectively. The plants were completely randomised within each block.

During the experiments, the nutrient solution circulated between the boxes and the accumulation tank. The composition of the modified Hoagland solution [Hoagland and Amon 1950, cited in 20] is shown in detail in Table 1. To ensure that the nutrients in the solution were available to the plants the pH and EC values of the solution were measured daily. If the pH deviated from 5.8 ± 0.25, it was adjusted by adding 4 M KOH or 4 M HCl, respectively. Plants were grown under high and low N supply solutions with 2.0 mM (i.e. 100% N = N100) and 0.5 mM (i.e. 25% N = N25) of NO_3_^-^, respectively. Cultivation of the plants lasted for 14 days in the hydroponic system with 16 h photoperiod, an irradiance of 300 μmol m^-2^ s^-1^ and day and night temperatures of 24°C and 16°C, respectively. To ensure that the roots received an ample supply of oxygen, air bubbles were constantly pressed with an air pump into the tank.

**Table 1:** Composition of the nutrient media for high (100% N = N100) and low (25% N = N25) N treatments

**Component**	**Concentration N100(in μM)**	**Concentration N25(in μM)**
Ca(NO_3_)_2_* 4H_2_O	2000.00	500.00
CaCl_2_	0.00	500.00
MES ^a^	2400.00	2400.00
K_2_SO_4_	700.00	700.00
MgSO_4_	500.00	500.00
KCl	100.00	100.00
KH_2_PO_4_	100.00	100.00
Na-EDTA ^b^	50.00	50.00
Fe(II)SO_4_* 7H_2_O	50.00	50.00
H_3_BO_3_	10.00	10.00
MnSO_4_ * H_2_O	0.50	0.50
CuSO_4_ * 5H_2_O	0.20	0.20
ZnSO_4_ * 7H_2_O	0.10	0.10
(NH_4_)_6_Mo_7_O_24_	0.01	0.01

^a^ 2-(N-morpholino)ethanesulfonic acid (MES) adjusted to pH 5.8.

^b^ Na-Ethylenediaminetetraacetic acid (EDTA).

**Figure 3:**
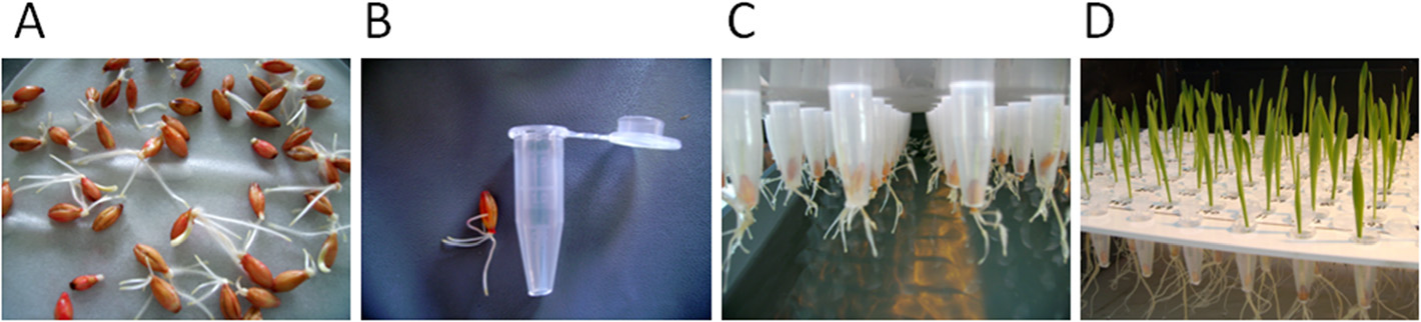
**Seed germination and plant cultivation. A**. Barley seeds were germinated in the dark at room temperature. **B**. After 72 h, seedlings that had three to five roots of 1.5 to 2.0 cm length and that had not yet started to extend the shoot were selected. Each seedling was inserted into a 1.5 ml reaction tube (Eppendorf, Hamburg) that was cropped at the bottom. **C**. The tubes with the seedlings were subsequently plugged into the perforated trays and placed on top of the boxes. **D**. Throughout the experiments the roots plunged into the nutrient solution whereas plant shoots grew on top of the tray.

For each experiment, seeds of ‘Scarlett’, ‘Barke’ and the S42ILs were coated with the fungicide Abavit UF® (BASF, Ludwigshafen). Seed germination and plant cultivation is shown in detail in Figure 3. To study the plant growth and responses to N deficiency on juvenile barley plants, ten traits were investigated. The methods of trait measurements and trait abbreviations are given in Table 2.

**Table 2:** List of ten quantitative traits investigated in the hydroponic system

**Abbr**.	**Trait**	**Method of measurement**	**Unit**
TN	Tiller number	Number of tillers with first leaf unfolded after 14 days	
LN	Leaf number	Number of leaves longer than 2 cm after 14 days	
LL	Leaf length	Length of the youngest completely unfolded leaf after 14 days	cm
HEI	Plant height	Length of the plant from basis to leaf tip after 14 days	cm
SDW	Shoot dry weight	Weight of shoot mass after 2 days of drying at 80°C	mg
RL	Root length	Length of the longest root from crown to root tip after 14 days	cm
RDW	Root dry weight	Root weight after 2 days of drying at 80°C	mg
LRR	Leaf to root ratio	Ratio of leaf to root length	
SRR	Shoot to root ratio	Ratio of shoot to root dry weight	
CC	Chlorophyll content	Average of 10 measurements with SPAD-502 meter ^a^ in the middle of the youngest leaf after 10 days	SPAD

^a^ SPAD 502 meter: Minolta Camera Co., Osaka, Japan.

### Statistical analyses

Analyses of data were carried out with SAS version 9.2 (SAS Institute 2010). Coefficients of broad sense heritability (*h*^2^, [29]) for the ten traits were estimated with S42IL data across treatments (ALL) as

(1) $$h^{2}=100\times \frac{V_{G}}{{\text{V}}_{G}+\frac{V_{\text{GxT}}}{t}+\frac{V_{\text{GxE}}}{e}+\frac{V_{\text{GxTxE}}}{\text{te}}+\frac{{\text{V}}_{\varepsilon }}{\text{ter}}}$$

and separately for N100 and N25 treatments, respectively, as

(2) $$h^{2}=100\times \frac{{\text{V}}_{G}}{\left(V_{G}+\frac{V_{\text{GxE}}}{e}+\frac{V_{\varepsilon }}{\text{er}}\right)}\text{,}$$

where V_*G*_, V_*GxT*_, V_*GxE*_, V_*GxTxE*_ and V_ε_ are the variance components genotype, genotype by treatment, genotype by experiment, genotype by treatment by experiment and the experimental error, with *t*, *e* and *r* being the number of treatments (*t* = 2), experiments (*e* = 6) and replications within an experiment (*r* = 6), respectively. Variance components were calculated with the VARCOMP procedure in SAS.

To calculate phenotypic correlations between the ten traits within a treatment and, in addition, within a trait between the two treatments, first the least squares means (LSMeans) were estimated for each S42IL with the MIXED procedure in SAS (see below). Subsequently, Pearson correlation coefficients (*r*) were calculated using the SAS procedure CORR.

We conducted the detection of significant line by trait associations for every trait revealing a heritability of *h*^2^ > 10% across treatments or within the N100 and N25 treatments, respectively. In this regard, a mixed model analysis of variance was carried out with the SAS procedure MIXED, either across treatments (ALL) as

(3) $$Y_{\mathrm{ijklmn}}=\mu +G_{i}+T_{j}+L_{k}+E_{l}\left(T_{j}\right)+B_{m}+G_{i}\times T_{j}+G_{i}\text{x}L_{k}+{\varepsilon_{n}}_{\left(ijklm\right)}\left(\text{Model}\;1\right)$$

or separately for the N100 and N25 treatment as

(4) $$Y_{\mathrm{iklmn}}=\mu +G_{i}+L_{k}+E_{l}+B_{m}+G_{i}\times \;L_{k}+\varepsilon_{n\left(iklm\right)}\left(\text{Model}\;2\right)$$

where μ is the general mean, *G*_*i*_ the fixed effect of the *i*th genotype (42 S42ILs plus ‘Scarlett’), *T*_*j*_ is the fixed effect of the *j*th treatment (N100 and N25), *L*_*k*_ is the random effect of the *k*th location (Kühnfeld and Heide), *E*_*l*_(*T*_*j*_) is the random effect of the *l*th experiment (1 to 6), nested within the *j*th treatment, *B*_*m*_ the random effect of the *m*th box (1 to 18), *G*_*i*_ × *T*_*j*_ is the fixed interaction effect of the *i*th genotype with the *j*th treatment, *G*_*i*_ × *L*_*k*_ is the random interaction effect of the *i*th genotype with the *k*th location and ε_*n*(*ijklm*)_ and ε_*n*(*iklm*)_ are the error terms of the phenotypes *Y*_*ijklmn*_ and *Y*_*iklmn*_, respectively, calculated from *n* replications (2 to 8) per box.

Afterwards a post-hoc Dunnett test was carried out to compare the LSMeans of the S42ILs with the LSMeans of the control genotype ‘Scarlett’ [30]. A line by trait association was accepted when the LSMeans of an S42IL differed significantly (*P* < 0.01) from ‘Scarlett’ across treatments (Model 1) and/or under the N100 and N25 treatment (Model 2), respectively. Significant line by trait associations were summarised to one QTL if the corresponding S42ILs (1) carried overlapping and/or flanking target introgressions and (2) showed genotypic effects of the same direction. The relative performance (RP) of an S42IL compared to the control genotype ‘Scarlett’ was calculated as

(5) RPIL=100×LSMeansS42IL−LSMeans'Scarlett'LSMeans'Scarlett',

where for each trait the LSMeans were calculated across both treatments or separately for N100 or N25, respectively.

## Results

### Trait performances and heritabilities of S42ILs

Table 3 indicates the trait performances of the S42IL population for ten studied traits in regard to the parameters mean, minimum, maximum, standard deviation (SD), coefficient of variation (CV) and heritability (*h*^2^). The values of each parameter are given across treatments and separately for the high and low N treatment. The high N treatment evoked an increase in the mean, minimum and maximum of the S42ILs for all traits except for root length. The highest CV was observed for root dry weight with 45.2% across both N levels. On the contrary, CVs for leaf length, plant height and chlorophyll content were below 20% across and within both N levels. Five traits showed higher heritability across treatments than per treatment, e.g. plant height with 80.8% across treatments versus 78.6% and 60.1% for the high and low N treatment, respectively. Of all investigated traits, root length showed the highest heritability (85.2% across N treatments). On the contrary, chlorophyll content was the trait that revealed the least heritability (0.0%, within N25).

**Table 3:** Parameters describing trait performances of the S42IL population in ALL, N100 and N25, respectively

**Trait**^**a**^		**Treatment**^**b**^	**N**^**c**^	**Mean**^**d**^	**Min**^**e**^	**Max**^**e**^	**SD**^**f**^	**CV**^**g**^	***h***^**2 h**^
TN		ALL	1379	1.5	1.0	4.0	0.6	39.9	31.1
		N100	922	1.7	1.0	4.0	0.6	35.5	52.9
		N25	457	1.1	1.0	3.0	0.3	30.5	11.9
LN		ALL	1379	4.0	2.0	8.0	1.1	26.4	21.2
		N100	922	4.2	2.0	8.0	1.1	26.7	37.8
		N25	457	3.6	2.0	6.0	0.8	21.0	12.1
LL	(in cm)	ALL	1379	24.3	11.1	33.1	3.3	13.5	75.4
		N100	922	24.8	11.1	33.1	3.2	13.0	73.0
		N25	457	23.4	11.3	30.2	3.2	13.6	47.5
HEI	(in cm)	ALL	1379	34.6	17.4	46.1	4.7	13.5	80.8
		N100	922	35.4	17.9	46.1	4.6	13.1	78.6
		N25	457	33.0	17.4	43.1	4.3	13.1	60.1
SDW	(in mg)	ALL	1379	117.8	15.0	249.0	43.7	37.1	7.3
		N100	922	133.1	15.0	249.0	40.9	30.7	3.3
		N25	457	87.0	15.0	190.0	31.2	35.9	32.6
RL	(in cm)	ALL	1379	34.3	7.6	61.0	8.8	25.6	85.2
		N100	922	33.3	7.6	58.1	9.3	27.9	78.9
		N25	457	36.3	15.0	61.0	7.2	19.7	73.4
RDW	(in mg)	ALL	1379	41.5	4.0	129.0	18.7	45.2	32.3
		N100	922	46.0	4.0	129.0	19.2	41.7	39.2
		N25	457	32.3	4.0	75.0	13.8	42.6	35.9
LRR		ALL	1379	0.8	0.4	2.6	0.2	31.2	72.4
		N100	922	0.8	0.4	2.6	0.3	32.8	48.8
		N25	457	0.7	0.4	1.1	0.1	18.7	78.4
SRR		ALL	1379	3.0	1.3	7.3	0.7	24.6	80.3
		N100	922	3.1	1.3	7.3	0.8	25.6	73.4
		N25	457	2.8	1.4	6.6	0.6	20.4	54.0
CC	(SPAD)	ALL	1377	36.4	20.8	51.8	6.5	17.8	3.1
		N100	922	37.3	21.3	51.8	6.2	16.6	2.0
		N25	455	34.5	20.8	48.9	6.6	19.2	0.0

^a^ Trait abbreviations are given in Table 2.

^b^ Treatment abbreviations.

ALL: across treatments, N100 and N25: high (100%) and low (25%) N supply.

^c^ Number of observations.

^d^ Average trait performance.

^e^ Minimum and maximum trait performance.

^f^ Standard deviation.

^g^ Coefficient of variation in %.

^h^ Heritability in %.

### Trait correlations

Pearson correlations between N treatments N100 and N25 were significant (*P* < 0.05) for all traits (diagonal in Table 4). Highly significant (*P* < 0.001) correlations between treatments were observed for the length traits leaf length, plant height, root length and both ratio traits. The highest correlation coefficient between treatments was found for root length (*r* = 0.70), whereas the correlation between N100 and N25 within tiller number was weak (*r* = 0.32).

**Table 4:** **Pearson correlation coefficients** (***r***) **between ten quantitative traits**, **calculated separately for treatment N25** (**bottom left triangle**) **and treatment N100** (**upper right triangle**) **and within trait between treatments N100 and N25** (**diagonal**), **based on LSMeans of 42 S42ILs**

**Trait**^**a**^	**TN**	^**b**^	**LN**		**LL**		**HEI**		**SDW**		**RL**		**RDW**		**LRR**		**SRR**		**CC**	
TN	**0**.**32**	*	0.74	***	0.55	***	0.49	***	0.61	***	0.07		0.53	***	0.23		−0.13		0.00	
LN	0.76	***	**0**.**38**	*	0.43	**	0.48	***	0.66	***	0.20		0.52	***	0.03		−0.04		0.01	
LL	0.21		−0.08		**0**.**64**	***	0.90	***	0.70	***	0.31	*	0.69	***	0.03		−0.34	*	0.25	
HEI	0.08		−0.07		0.91	***	**0**.**68**	***	0.75	***	0.53	***	0.81	***	−0.02		−0.47	**	0.40	**
SDW	0.48	***	0.44	**	0.55	***	0.63	***	**0**.**44**	**	0.37	*	0.54	***	−0.14		0.03		0.45	**
RL	0.11		0.25		0.41	**	0.43	**	0.44	**	**0**.**70**	***	0.60	***	−0.89	***	−0.47	**	0.39	**
RDW	0.13		0.07		0.55	***	0.59	***	0.78	***	0.43	**	**0**.**45**	**	−0.28		−0.79	***	0.53	***
LRR	−0.10		−0.29		0.16		−0.11		−0.03		−0.82	***	−0.25		**0**.**61**	***	0.34	*	−0.32	*
SRR	0.16		0.22		−0.34	*	−0.34	*	−0.08		−0.28		−0.66	***	0.25		**0**.**64**	***	−0.36	*
CC	0.42	**	0.40	**	0.35	*	0.48	**	0.63	***	0.39	**	0.58	***	−0.20		−0.15		**0**.**39**	**

^a^ Trait abbreviations are given in Table 2.

^b^ Significant correlation coefficients are indicated with * *P* < 0.05, ** *P* < 0.01 and *** *P* < 0.001.

When comparing the correlation matrices of both N treatments, pairwise correlations among traits showed a higher number of significant (*P* < 0.05) correlations within N100 (31, upper right triangle in Table 4) compared to N25 (24, lower left triangle in Table 4). For example the correlation between tiller number and plant height was highly significant at N100 (*r* = 0.49 with *P* < 0.001) but absent at N25 (*r* = 0.08 with *P* > 0.05). The highest correlation coefficients were found between traits that belong to the same trait complex like leaf length and plant height (*r* = 0.90 within N100) or root length and leaf to root ratio (*r* = -0.89 within N100). No correlation was found between tiller number and chlorophyll content (*r* = 0.00 within N100). The trait leaf to root ratio showed the lowest number of significant correlations to the other traits. Only correlations to root length and shoot to root ratio were significant within N100.

### QTL detection

With regard to the trait heritabilities, QTL detection was carried out for all traits except for SDW (ALL and N100) and CC (ALL, N100 and N25), revealing trait heritabilities below 10%. In total, the post-hoc Dunnett test revealed 126 significant (*P* < 0.01) line by trait associations for nine traits. Fifty-six associations (44%) were highly significant (*P* < 0.001). As presented in Table 5, 43 associations were found across treatments (Model 1), 41 under N100 and 42 under N25 (Model 2), respectively. S42IL-143, -133 and -135, with target introgressions on chromosomes 1HL, 7HS and 7HL, respectively, revealed the highest number of trait associations (twelve, ten and ten associations, respectively).

**Table 5:** **List of 58 significant** (***P*** <**0**.**01**) **QTL effects among nine traits and 42 S42ILs**

**Trait**^**a**^	**QTL name**	**Line**	**Chr.**^**b**^	**Target intro.**^**b**^**(cM)**	**Effect**	**LSMeans S42IL**^**d**^	**LSMeans Scarlett**^**d**^	**Diff**^**e**^	**RP(IL)**^**f**^**(in %)**	**QTL effect in S42 or S42IL**^**g**^	**Candidate gene**^**h**^
TN	QTn.S42IL-1H	S42IL-101	1H	001.1 - 013.5	N100	2.0	1.6	0.4	22.9		
	QTn.S42IL-4H	S42IL-123	4H	128.9 - 172.3	ALL+N100	1.7	1.4	0.4	26.4*	I, V	
	QTn.S42IL-6H	S42IL-148	6H	003.3 - 010.7	N100	2.1	1.6	0.5	30.7*		
	QTn.S42IL-7H	S42IL-135	7H	101.2 - 152.3	N100	2.1	1.6	0.4	25.5		
		S42IL-136	7H	134.4 - 152.3	N100	2.0	1.6	0.4	22.7		
LN	QLn.S42IL-2H	S42IL-109	2H	064.0 - 110.8	N25	4.2	3.2	0.9	28.3		*Eam6*^1^, *Flt*-*2L*^2^
	QLn.S42IL-4H	S42IL-123	4H	128.9 - 172.3	ALL	4.3	3.6	0.8	21.3		
		S42IL-124	4H	171.3 - 183.5	ALL+N100	4.2	3.6	0.7	19.1		*VRN*-*H2*^3^
	QLn.S42IL-6H	S42IL-148	6H	003.3 - 010.7	N100	4.7	3.9	0.8	21.0		
	QLn.S42IL-7H.a	S42IL-133	7H	017.3 - 051.9	ALL	4.3	3.6	0.7	19.6		*VRN*-*H3*^4^
	QLn.S42IL-7H.b	S42IL-135	7H	101.2 - 152.3	N100	4.6	3.9	0.7	18.8		
	QLn.S42IL-7H.c	S42IL-139	7H	198.7 - 229.7	N25	4.1	3.2	0.9	27.5		
LL	QLl.S42IL-1H	S42IL-143	1H	130.7 - 173.5	ALL+N100+N25	20.3	23.7	−3.4	−14.3*		*HvFT3*^5^
	QLl.S42IL-2H	S42IL-107	2H	034.3 - 066.8	N100	22.9	25.3	−2.4	−9.3		
		S42IL-108	2H	034.3 - 104.8	N100	22.8	25.3	−2.5	−9.7		
	QLl.S42IL-3H.a	S42IL-113	3H	204.5 - 239.7	N100	23.0	25.3	−2.3	−9.1		*sdw1*^6^
	QLl.S42IL-3H.b	S42IL-115	3H	204.5 - 255.1	N25	25.4	22.1	3.3	15.0		*sdw1*^6^
	QLl.S42IL-4H	S42IL-121	4H	074.1 - 119.1	N25	24.7	22.1	2.6	11.8		
		S42IL-123	4H	128.9 - 172.3	ALL+N25	26.0	23.7	2.3	9.7*		
	QLl.S42IL-5H	S42IL-126	5H	145.6 - 200.1	N25	25.1	22.1	3.1	13.8		*ari*-*e*.*GP*^7^
	QLl.S42IL-6H	S42IL-131	6H	140.0 - 180.7	N100	22.9	25.3	−2.4	−9.5		*cul2*^8^
	QLl.S42IL-7H.a	S42IL-133	7H	017.3 - 051.9	N100	22.6	25.3	−2.6	−10.4*		*brh1*^9^
		S42IL-134	7H	051.9 - 107.4	ALL+N100	21.6	23.7	−2.0	−8.6*		*brh1*^9^
	QLl.S42IL-7H.b	S42IL-135	7H	101.2 - 152.3	ALL+N25	25.6	23.7	1.9	8.0		
		S42IL-136	7H	134.4 - 152.3	N25	26.3	22.1	4.2	18.9*		
		S42IL-137	7H	134.4 - 193.9	ALL+N25	26.1	23.7	2.5	10.4*		
	QLl.S42IL-7H.c	S42IL-138	7H	176.4 - 229.7	ALL+N25	25.6	23.7	1.9	8.1*		
HEI	QHei.S42IL-1H.a	S42IL-102	1H	001.1 - 098.2	ALL	35.3	33.0	2.3	7.0	IV	
	QHei.S42IL-1H.b	S42IL-143	1H	130.7 - 173.5	ALL+N100+N25	27.0	33.0	−6.0	−18.1*	I, III, V	*HvFT3*^5^
	QHei.S42IL-2H.a	S42IL-106	2H	022.4 - 034.3	N25	34.5	30.6	3.9	12.9	V	
	QHei.S42IL-2H.b	S42IL-109	2H	064.0 - 110.8	ALL+N25	35.3	33.0	2.3	6.9	I - V	*Eam6*^1^, *Flt*-*2L*^2^
		S42IL-110	2H	102.7 - 104.8	N25	34.3	30.6	3.7	12.1	I, III, IV, V	*Flt*-*2L*^2^
	QHei.S42IL-3H	S42IL-115	3H	204.5 - 255.1	ALL+N25	36.6	33.0	3.6	10.9*	I, IV, V	*sdw1*^6^
	QHei.S42IL-4H	S42IL-121	4H	074.1 - 119.1	ALL+N25	35.4	33.0	2.4	7.3*	II, V	
		S42IL-123	4H	128.9 - 172.3	ALL+N25	35.5	33.0	2.6	7.8*	V	
	QHei.S42IL-5H	S42IL-126	5H	145.6 - 200.1	N25	34.6	30.6	4.1	13.3		*ari*-*e*.*GP*^7^
	QHei.S42IL-6H	S42IL-148	6H	003.3 - 010.7	ALL	35.4	33.0	2.5	7.5		
	QHei.S42IL-7H.a	S42IL-133	7H	017.3 - 051.9	ALL+N100	30.0	33.0	−3.0	−9.1*		*brh1*^9^
		S42IL-134	7H	051.9 - 107.4	N100	30.9	35.4	−4.5	−12.8*	I - V	*brh1*^9^
	QHei.S42IL-7H.b	S42IL-135	7H	101.2 - 152.3	ALL+N25	36.1	33.0	3.2	9.6*	I, IV, V	
		S42IL-136	7H	134.4 - 152.3	ALL+N25	35.7	33.0	2.8	8.4*	I, V	
		S42IL-137	7H	134.4 - 193.9	ALL+N25	37.2	33.0	4.2	12.8*	I, II, V	
	QHei.S42IL-7H.c	S42IL-138	7H	176.4 - 229.7	ALL+N25	35.9	33.0	2.9	8.9*		
		S42IL-139	7H	198.7 - 229.7	N25	34.5	30.6	3.9	12.9		
SDW	QSdw.S42IL-3H	S42IL-115	3H	204.5 - 255.1	N25	104.1	65.8	38.3	58.2	I	
	QSdw.S42IL-4H	S42IL-121	4H	074.1 - 119.1	N25	100.7	65.8	34.9	53.0	I, VI	
	QSdw.S42IL-7H	S42IL-136	7H	134.4 - 152.3	N25	101.1	65.8	35.3	53.6	VI	
		S42IL-137	7H	134.4 - 193.9	N25	102.0	65.8	36.2	55.0	VI	
RL	QRl.S42IL-1H.a	S42IL-141	1H	094.9 - 127.7	ALL+N100	37.3	33.0	4.3	13.0		
	QRl.S42IL-1H.b	S42IL-143	1H	130.7 - 173.5	ALL+N100	27.9	33.0	−5.1	−15.5*		
	QRl.S42IL-1H.c	S42IL-142	1H	188.5 - 205.1	N25	40.0	33.8	6.2	18.2	VII	
	QRl.S42IL-2H	S42IL-106	2H	022.4 - 034.3	ALL+N100	37.8	33.0	4.8	14.6		
		S42IL-107	2H	034.3 - 066.8	ALL+N100+N25	39.3	33.0	6.3	19.0*		
	QRl.S42IL-3H	S42IL-113	3H	204.5 - 239.7	N25	40.2	33.8	6.3	18.7		*sdw1*^6^
	QRl.S42IL-4H.a	S42IL-121	4H	074.1 - 119.1	N25	41.1	33.8	7.2	21.4*		
	QRl.S42IL-4H.b	S42IL-124	4H	171.3 - 183.5	ALL+N100+N25	38.7	33.0	5.6	17.0*		
	QRl.S42IL-5H	S42IL-126	5H	145.6 - 200.1	N100	25.5	32.2	−6.7	−20.8*		*ari*-*e*.*GP*^7^
	QRl.S42IL-6H.a	S42IL-148	6H	003.3 - 010.7	ALL+N25	38.1	33.0	5.0	15.2*		
	QRl.S42IL-6H.b	S42IL-128	6H	071.4 - 132.2	ALL+N100+N25	39.9	33.0	6.9	20.9*		
		S42IL-129	6H	073.9 - 133.5	ALL+N100+N25	38.4	33.0	5.4	16.4*		
		S42IL-130	6H	098.7 - 180.7	ALL+N100+N25	39.5	33.0	6.5	19.7*		
	QRl.S42IL-7H.a	S42IL-133	7H	017.3 - 051.9	ALL+N100+N25	27.2	33.0	−5.8	−17.7*		*brh1*^9^
		S42IL-134	7H	051.9 - 107.4	ALL	28.1	33.0	−5.0	−15.1		*brh1*^9^
	QRl.S42IL-7H.b	S42IL-135	7H	101.2 - 152.3	ALL+N100+N25	41.5	33.0	8.5	25.7*		
RDW	QRdw.S42IL-1H.a	S42IL-102	1H	001.1 - 098.2	ALL+N100	45.1	33.9	11.2	33.0		
	QRdw.S42IL-1H.b	S42IL-143	1H	130.7 - 173.5	N100	31.2	43.5	−12.3	−28.4		
	QRdw.S42IL-2H	S42IL-109	2H	064.0 - 110.8	ALL+N100+N25	48.1	33.9	14.2	41.9*		
LRR	QLrr.S42IL-2H	S42IL-107	2H	034.3 - 066.8	ALL+N100	0.6	0.8	−0.2	−23.7*		
	QLrr.S42IL-5H	S42IL-126	5H	145.6 - 200.1	ALL+N100	0.9	0.8	0.2	24.1*		*ari*-*e*.*GP*^7^
	QLrr.S42IL-6H	S42IL-128	6H	071.4 - 132.2	ALL+N100	0.6	0.8	−0.2	−20.9*		
	QLrr.S42IL-7H	S42IL-135	7H	101.2 - 152.3	N100	0.7	0.8	−0.2	−21.0		
SRR	QSrr.S42IL-1H	S42IL-143	1H	130.7 - 173.5	ALL+N100+N25	3.9	2.9	1.0	35.1*		
	QSrr.S42IL-2H	S42IL-107	2H	034.3 - 066.8	ALL+N100	3.4	2.9	0.5	16.3		
	QSrr.S42IL-4H	S42IL-123	4H	128.9 - 172.3	N100	3.6	3.0	0.6	18.4*		
	QSrr.S42IL-5H	S42IL-126	5H	145.6 - 200.1	ALL+N100	3.3	2.9	0.4	13.5*		*ari*-*e*.*GP*^7^
	QSrr.S42IL-7H	S42IL-133	7H	017.3 - 051.9	ALL+N100+N25	3.8	2.9	0.9	30.2*		

^a^ Trait abbreviations are given in Table 2.

^b^ Chromosomal location and extent (in centiMorgan) of the target introgression according to Schmalenbach et al. [19].

^c^ Significant (*P* < 0.01) line effect across treatments (ALL), under high N treatment (N100) or under low N treatment (N25), determined by the Dunnett test.

^d^ Least squares means of the S42IL and 'Scarlett', respectively. If the S42IL showed a significant association across treatments, the LSMeans across treatments (ALL) is given. If the S42IL showed a significant association solely in N100 or in N100 and N25, respectively, the LSMeans of N100 is given. If the S42IL showed a significant effect solely under N25, the LSMeans of N25 is given.

^e^ Difference between the LSMeans of the S42IL and the LSMeans of 'Scarlett'.

^f^ Relative trait performance of the S42IL compared to 'Scarlett', calculated as RP(IL) = 100 × [LSMeans(S42IL) – LSMeans(‘Scarlett’)]/LSMeans(‘Scarlett’). Highly significant (*P* < 0.001) trait associations of a QTL are labelled with an asterisk.

^g^ Corresponding QTL in S42 or S42IL published in: I von Korff et al. [45], II Schmalenbach et al. [14], III Wang et al. [18], IV von Korff et al. [44], V Saal et al. [22], VI Schnaithmann and Pillen [23], VII Naz et al. [16].

^h^ Candidate genes published in: 1 Franckowiak and Konishi 1996, cited in [46], 2 Chen et al. [47], 3 Laurie et al. [49], 4 Yan et al. [48], 5 Faure et al. [58], 6 Laurie et al. [54], 7 Chloupek et al. [56], 8 von Wettstein 1993, cited in [61], 9 Li et al. [57].

With regard to the position and extent of the corresponding target introgressions, all associations were summarised to 58 QTL. Among the nine traits, root length, plant height and leaf length showed the highest number of QTL effects (twelve, eleven and ten QTL, respectively). On the contrary, root and shoot dry weight were the traits that revealed only three and two QTL effects, respectively. For 43 QTL (74%), the *Hsp* allele showed an increasing trait effect compared to ‘Scarlett’. In the following paragraphs, the results shown in Table 5 are described in detail for each of the nine traits.

### Shoot traits

A total of five S42ILs showed significant line by trait associations for tiller number (TN). The associations were summed up to four QTL, located on chromosomes 1H, 4H, 6H and 7H. At QTn.S42IL-4H the line effects were detected across treatments whereas the other QTL showed only effects under N100. All QTL were due to a higher trait performance of the S42IL compared to ‘Scarlett’, with a maximum increase of 0.5 more tillers per plant (30.7%) by S42IL-123 across treatments (QTn.S42IL-6H).

For leaf number (LN), seven S42ILs significantly differed from ‘Scarlett’. The effects were summarised to six QTL, which were located on chromosomes 2H, 4H, 6H and 7H. Two QTL each were detected across treatments, under N100 and under N25, respectively. At all QTL the S42ILs showed an increase in LN. S42IL-109 showed the maximum difference to ‘Scarlett’ at QLn.S42IL-2H with 0.9 (28.3%) more leaves per plant under N25.

There were 15 S42ILs that were significantly associated to leaf length (LL). The associations were subsequently summarised to 10 QTL. Target introgressions of the corresponding S42ILs were located on all seven barley chromosomes. Two and three QTL showed significantly different trait performances of the S42IL solely under N25 and N100, respectively. Five QTL included lines with relatively shorter leaves than Scarlett. At QLl.S42IL-1H.b, S42IL-143 revealed the maximum decreasing effect compared to ‘Scarlett’ under N100 with -3.4 cm (-14.3%). The *Hsp* introgression of S42IL-136 caused the maximum elongation of the youngest leaf with 4.2 cm (18.9%) at QLl.S42IL-7H.b.

The Dunnett test revealed 17 lines that were significantly associated with plant height (HEI). Altogether eleven QTL from all seven barley chromosome were defined. One QTL effect was only found under N25. Two QTL were due to relatively lower plant height of the corresponding S42ILs. As shown for LL, S42IL-143 revealed the maximum decreasing effect compared to ‘Scarlett’ across treatments with -6.0 cm (-18.1%, QHei.S42IL-1H.b). At QHei.S42IL-7H.c, S42IL-126 had the maximum increasing effect compared to ‘Scarlett’ under N25 with 4.1 cm (13.3%).

Due to low heritabilities with *h*^2^ < 10% (see Table 3), QTL detection for shoot dry weight (SDW) was only conducted within the N25 treatment. There, four S42ILs differed significantly from ‘Scarlett’. The effects were summarised to three QTL, which were located on chromosomes 3H, 4H and 7H. The QTL showed an increase in trait performance of the S42ILs revealing an up to 58.2% higher SDW.

### Root traits

A total of 16 lines showed significantly different root lengths (RL) compared to ‘Scarlett’. The associations were summed up to twelve QTL, located on all barley chromosomes. Three QTL effects, on chromosomes 1H, 2H and 3H, were only found under N25. At QRl.S42IL-5H the strongest decreasing QTL effect was found for S42IL-126 under N100 with -6.7 cm (-20.8%). S42IL-121 showed the strongest increasing QTL effect at QRl.S42IL-4H.a with a 7.2 cm (21.4%) longer root compared to ‘Scarlett’.

Three S42ILs showed significant line by trait associations for root dry weight (RDW). According to this, three QTL were defined, located on chromosomes 1H and 2H. At QRdw.S42IL-1H.b the line effect was solely present under N100. This QTL caused a lower trait performance of S42IL-143 compared to ‘Scarlett’, with a decrease of -12.3 mg (-28.4%). The maximum increase was found at QRdw.S42IL-2H with a plus of 14.2 mg (41.9%).

### Shoot to root trait ratios

For leaf to root ratio (LRR), four S42ILs differed significantly from ‘Scarlett’. These effects corresponded to four QTL, located on chromosomes 2H, 5H, 6H and 7H. QLrr.S42IL-7H was only detected under N100. The QTL on chromosome 5H, QLrr.S42IL-5H, was the only one with an increase in the trait performance by 0.2 (24.1%). S42IL-107 showed the maximum decline compared to ‘Scarlett’ at QLrr.S42IL-2H with -0.2 (-23.7%).

A total of five S42ILs showed a significantly different trait performance from ‘Scarlett’ for shoot to root ratio (SRR). The effects corresponded to five QTL, located on chromosomes 1H, 2H, 4H, 5H and 7H. QSrr.S42IL-4H was only detected under N100. All QTL revealed an increase in trait performance of the S42ILs. S42IL-143 showed the maximum increase compared to ‘Scarlett’ at QSrr.S42IL-1H with 1.0 (35.1%).

### Chlorophyll content

Due to low heritabilities with *h*^*2*^ < 10% (see Table 3), no QTL detection was carried out for chlorophyll content (CC).

## Discussion

### Suitability of the hydroponic system for early selection of favourable S42ILs

In 2008 and 2011, a set of 42 introgression lines was tested in six hydroponic experiments under two different N treatments. The fixation of the plants in Eppendorf tubes during growth in the hydroponic system allowed us to analyse shoots and roots simultaneously. Huang et al. [31], who used a similar plant fixation in their hydroponic system, also highlighted the flexibility of those systems. Also other studies reported that plant responses to low N availability vary among genotypes, developmental stages and plant organs [32]. Regarding the N treatments, we did not observe any symptoms of starvation on the plants under full supply condition, which indicates that the macro and micro nutrients were sufficiently supplied throughout the experiment [33]. In contrast, the reduction of N supply caused a severe reaction in most traits studied. For example, root length was increased while plant height was decreased under N deficiency (Table 3).

Taking the parameter heritability as a quality measure of genetic to phenotypic variation, we found that the phenotypic data collected in our hydroponic system predominantly revealed strong and medium heritabilities with *h*^2^ > 70% for five traits and 70% >*h*^2^ > 30% for two traits, respectively. Shoot dry weight (ALL and N100) and chlorophyll content (ALL, N100 and N25) have to be considered as not heritable with *h*^2^ > 10% (Table 3). In this regard, the mixed model analysis of variance was not carried out for the respective treatments of those traits.

For the other traits, the phenotypic data collected from the hydroponic experiments were subsequently analysed in a line by trait association study. In total, 12 out of 58 QTL effects (21%) that we found with juvenile S42ILs in the hydroponic system corresponded to QTL for comparable traits that were detected in previous field and greenhouse studies with adult plants of a similar S42IL set or with the original S42 population (Table 5). The corresponding QTL predominantly controlled the traits plant height (8 x) and, to a lesser extent, tiller number (1 x) and root length (1 x). Both findings, the medium to strong heritabilities observed and the high amount of corresponding QTL, support the idea to use the hydroponic system for early mapping of QTL and early selection of the QTL-bearing genotypes in plant physiology experiments and in plant breeding.

In the following paragraphs, we discuss the detection of juvenile plant growth QTL in wild barley ILs in general and in regard to N deficiency. Finally, we will draw connections between our QTL results, candidate genes and previously described QTL.

### Detection of juvenile plant development QTL

The major advantage of growing juvenile barley plants is the fast collection of phenotypic data, for instance within 14 days in our hydroponic system. This time-, cost- and effort-reducing aspects are of major importance for plant breeding where the reduction of time for phenotypic assessment can substantially improve the breeding process because new varieties may be introduced earlier and at lower selection costs to the market. Furthermore, the hydroponic system allows to simultaneously study the development of barley root and shoot traits [32]. Mattsson et al. [34], for instance, could show that in barley root relative growth rate increased while root dry weight decreased at the same time. In addition, the reaction of nutrient deficiencies on barley growth has often been carried out with juvenile plants [24,27,35]. We also conducted a juvenile hydroponic experiment to map QTL that control barley root and shoot traits in regard to N supply.

With 126 significant (*P* < 0.01) line by trait associations for nine traits under study, the number of QTL detected in the hydroponics experiment was higher than in any other study that was carried out with adult plants of the S42IL population. For example, Schmalenbach et al. [14] found 65 associations for seven field agronomical traits in the S42IL population. The increase in the number of associations may be attributed to the different screening methods (hydroponic vs. field testing), the different developmental stages (juvenile vs. adult plants), different traits (shoot and root vs. agronomic traits) and the varying N levels, as already reported for the S42 population by Saal et al. [22].

The highest number of QTL was found for the length parameters leaf length, plant height and root length. The three traits showed high heritabilities of 75.4%, 80.8% and 85.2% across both treatments, respectively. Trait heritability is known to influence the number and the probability of detected QTL. For traits with heritabilities of *h*^2^ < 10%, Ellis et al. [35] did not detect any QTL.

The QTL we mapped were scattered across the whole genome (Figure 1). We detected the highest number of QTL effects on chromosome arm 7HL. Here, QTL were found for all traits except root dry weight and shoot to root ratio (Figure 1). In the same chromosomal region QTL effects for plant height and shoot dry weight where already described in studies of the S42IL and S42 population (Table 5). In this regard, genes located on chromosome arm 7HL may be particularly important for regulation of plant growth.

At 75% of all detected QTL, the S42ILs showed increasing *Hsp* effects (Table 5). In contrast, when studying agronomic traits with S42ILs in the field, only 47% of the detected associations showed increasing *Hsp* effects [14]. Since most of the traits measured are related to a fast growth during the juvenile phase, this finding was expected. It may be explained by the fact that the donor of the S42ILs, the *Hsp* accession ‘ISR42-8’, is well adapted to survival under low nutrient supply. Those S42ILs that reveal QTL effects compared to the elite barley cultivar ‘Scarlett’ may have, thus, contributed exotic *Hsp* alleles that speed up or increase juvenile growth. Under high N treatment an increase of the traits we evaluated is generally desirable.

A fast juvenile plant development is of great importance, especially when plants are grown under field conditions. Baethgen et al. [24] reported that the application of a high dosage of N early in the growing season stimulated tiller formation of malting barley. However, many of these tillers did not develop fertile ears and, thus, the extra dosage of N fertilizer was wasted. In addition, for malting barley one has to consider that a high N supply during grain filling may be conflicting with grain quality [25]. On the other hand, if plants are cultivated under low N supply, many tillers may also be undesirable since plants may not be able to produce ears and fill the grains for all of the tillers. Multivariate analyse methods like the principle component analysis, are important statistical tools for primary assessment of data structure and, in addition, may support to identify the most important traits for phenotypic selection. However, our primary aim was to locate QTL early on under control and N starvation conditions in a hydroponic system. Thus, the difference in trait performance of the S42ILs compared to ‘Scarlett’ was of greatest interest to us. Due to space and time limitations, a thorough multivariate analysis will be delayed to a follow up study where the most promising traits and, in addition, favourable QTL alleles, will be used for genotype selection. Subsequently, the potential selection gain will be measured within the selected offspring generation.

### QTL and N deficiency

Twenty-five of the 42 associations we found under N25 (Model 2) were simultaneously present across treatments (Model 1, see Table 5). Jana and Wilen [36] recommended that it is optimal, when lines that are good under non stress conditions, also perform well under stress conditions. This may be of great importance for breeders, who do not have to conduct two parallel breeding programs, and for farmers, who do not have to select different varieties based on expected N stress conditions. On the other hand, selection of breeding lines in stress environments may result in genetic gains by using adapted germplasm, especially in regard to originally low-input crops like malting barley [37]. However, our results indicated that a number of QTL effects were not in common between both N treatments. Sixteen and 17 QTL were solely detected under N100 or N25, respectively. In one case, the direction of the exotic QTL effect was actually opposed, decreasing leaf length under high N supply while increasing leaf length under low N supply (see Table 5, QLl.S42IL-3H.a and QLl.S42IL-3H.b, present in S42IL-113 and-115, respectively). We, thus, conclude that it may be worth to select barley cultivars for N stress tolerance separately from experiments under low N and high N fertilization.

Associations to tiller number and leaf number were detected across treatments, under N100 and under N25. Similar to our results, Andersen [38] also found an increase in the number of leaves under low N treatment in juvenile and adult plants. For leaf length, two QTL where found in the same region of chromosome 3H. The decreasing effect at QLl.S42IL-3H.a and the increasing effect at QLl.S42IL-3H.b were detected under N100 and N25, respectively. Our results suggest that the gene effect on reducing leaf length is reversed under N deficiency. Shoot dry weight of the S42ILs was increased by up to 58.2% under N25 (Table 3), which was already observed by Marshall and Ellis [25] (Table 5).

Regarding plant development, root characteristics are of great importance, which is especially shown when plants are grown under stress conditions [39]. Most root length QTL showed an increase of trait performance under N25, which may indicate that the S42ILs try to react to N starvation by increasing their capacity to take up N from the solution. In contrast, root dry weight showed a mean reduction of approximately 30% compared to the control treatment, which was also shown in various other studies [28,40], and only two increasing QTL were found for this trait. In addition, Karley et al. [27] demonstrated in barley that trait differences between the N treatments increased with advancing growth stages, except for root parameters. The authors reported a lack of genotype by N supply interaction for root traits and assumed a limited potential for exploiting genetic variation to improve barley root performance [27]. However, this finding is in contrast to our study, where we detected a substantial number of root-related QTL effects. Also Naz et al. [16] reported a severe *Hsp* QTL effect on chromosome 5H that is present in a S42IL and caused an substantial increase in root biomass, both, under drought and controlled water conditions. Unfortunately, this particular line, S42IL-176, was not included in the set of S42ILs that we studied under hydroponics.

We did not detect leaf to root ratio or shoot to root ratio QTL solely under low N supply. Also Bahrman et al. [32] did not detect variety by N level effects in the shoot to root ratio of eight week old wheat plants. We conclude that in order to detect a change in shoot to root ratio, plants may have to be under stress for a longer time [41].

Because of the missing heritability for chlorophyll content we did not conduct the analysis of variance for this trait. In our experiments genetic effects on chlorophyll content were, thus, not stable across or within treatments. This finding is in accordance with Cartelat et al. [42], who reported that the change in chlorophyll content was reflecting the N nutrition status, but showed no direct association to genotype, growth stage or environment, respectively.

### Comparison of our results with known candidate genes and QTL

As highlighted before, we revealed more QTL than in any other QTL study conducted with the S42IL or S42 populations, indicating that new genes may have been expressed in our hydroponic study. Furthermore, comparing trait performances of coinciding S42ILs, analysed simultaneously in our study and in field and greenhouse studies [14,23], revealed low correlations, for example between juvenile and adult plant height (*r* < 0.08, data not shown) as well as between juvenile and adult number of tillers and ears, respectively (*r* < 0.12, data not shown). However, in Table 5 altogether 12 QTL for tiller number, plant height, shoot dry weight and root length under hydroponics corresponded to previously detected field and greenhouse QTL for number of ears, plant height, biomass and root length [14,16,18,22,23,43,44]. It remains open if the same genes in the respective S42ILs caused the QTL effects under hydroponic and field or greenhouse cultivation. Alternatively, it may also be possible that linked *Hsp* alleles that are present on the same introgression may have caused the QTL effects in the independent studies. To further elaborate on this question, interesting QTL effects should be narrowed down and validated in high-resolution offspring lines that are derived from the original S42ILs and segregate for the detected QTL effects [19].

In the following paragraphs, we attempt to draw connections between 12 significant *Hsp* effects and already described candidate genes and QTL effects.

Andersen [38] reported that juvenile tiller number is highly correlated with adult plant ear number, which is also an indicator for yield potential. We mapped one tiller number QTL on chromosome 4H that was consistent with two previously detected QTL for number of ears in population S42 [22,45]. At QTn.S42IL-7H, S42IL-135 revealed a strong increase in tiller number. Ellis et al. [35] also found a strong increasing effect on tiller number in the same region of 7HL with barley seedlings, too.

The number of leaves influences the photosynthetic capacity of the plant. Thus, growth, development and yield may be co-regulated through the control of leaf number. Cuesta-Marcos et al. [46] estimated the number of leaves until heading in barley lines and found a significant QTL effect nearby the earliness per se locus *Eam6* [Franckowiak and Konishi 1995, cited in 46]. We also found a QTL in this chromosomal region with S42IL-109 under N25 at QLn.S42IL-2H. In addition, the *Flt2L* locus, controlling flowering time and plant height, maps to the same region [47] and may be present in S42IL-110. Furthermore, *VRNH2* and *VRNH3*, two major genes determining the requirement for vernalisation, map to the same chromosomal regions where we found leaf number QTL in the S42IL population [18,48-50]. Their influences on growth related traits [18,51] may be due to primary effects on juvenile leaf number as it was already shown for the final leaf number in wheat [52].

We detected one decreasing and one increasing *Hsp* effect for leaf length on chromosome 3H under N100 and N25, respectively. Similar to this, Gregory et al. [53] described a reduction in barley shoot length under control conditions and associated the effect with the candidate gene for dwarfism on chromosome 3HL: *sdw1*[54,55]. Saal et al. [22] also detected two *Hsp* effects on total plant height under low and high N treatment on 3HL, but there was no difference between the directions of both effects. Thus, we conclude that *sdw1* controlled juvenile shoot development in our study; however, plant reaction depends on the status of N supply. On chromosomes 5H and 7H two additional dwarfing genes are located: *arie*.*GP* and *brh1*. At both loci, we also detected QTL for leaf length. Chloupek et al. [56] already described that *arie*.*GP* has pleiotropic effects on plant growth in general, including shoot development. However, the *arie*.*GP* allele is a very rare allele in the elite barley gene pool, making it unlikely that ‘Scarlett’ carries this dwarfing gene. In addition, *brh1*[57] was associated with strong effects on plant height and leaf length in a barley backcross population [51]. S42IL-133 and -134, probably carrying *brh1*, showed *Hsp* effects of the same direction on leaf length.

We verified 33 QTL, previously described for adult plant height in studies with the S42 or S42IL population, with twelve lines associated to juvenile plant height (Table 5). In addition, we found candidate genes influencing barley shoot development, which may explain the *Hsp* effects. On chromosome 1H for example, S42IL-143 caused the strongest decreasing effect on plant height. QTL of the same direction were described with the S42 population [22,45] and the decreasing *Hsp* effect on adult plant height at the *HvFT3* locus [58] on chromosome 1HL was already described [18]. Besides, the three dwarfing genes on chromosomes 3H, 5H and 7H revealed strong effects on plant height in our experiments. The effects are similar to those described for leaf length and are discussed in detail in the previous paragraph. Additionally, for each of the three dwarfing genes we refer to *Hsp* effects described in previous studies carried out with adult plants of the S42IL or S42 population.

On chromosome 4H, an increasing *Hsp* effect on straw weight was described by Schnaithmann and Pillen [23] which is consistent with our effect QSdw.S42IL-4H. Von Korff et al. [45] also detected a biomass effect on chromosome 4H.

Semi-dwarf genes are known to have a major impact on aerial plant organs [54,55] but their effects on root traits was also affirmed in the literature [56]. In this regard, Wojciechowski et al. [59] found differences in wheat root length due to the dwarfing-gene *Rht*. With our data, we found two contrasting root length QTL in the chromosomal regions of the barley semi-dwarfing genes *sdw1* and *arie*.*GP* on chromosomes 3H and 5H, respectively. At both loci, contrasting effects for several shoot and root parameters like stabile isotope discrimination for C and N were reported [35,56]. We assume that the *Hsp* alleles at the dwarfing-gene loci may also have caused the root length QTL.

The number of QTL effects detected for the leaf to root ratio was relatively low compared to the traits it was derived from. Wang et al. [60] demonstrated that QTL for mathematically derived traits are harder to detect than the component traits they are derived from.

## Conclusion

In general, the verification of a QTL is interpreted as the stability of its effect across genotypes or environments. As our data suggest, the verification of a QTL may also be interpreted as the stability of its effect across developmental stages. In total, 12 QTL effects we reported for juvenile S42ILs in the hydroponic system corresponded to QTL previously described in field and greenhouse studies conducted with the S42ILs or the parent population S42. S42IL-135, -136 and -137 revealed multiple increasing *Hsp* effects on the long arm of chromosome 7H. So far, we could not associate the QTL with any candidate gene. We therefore aim for further studies with high-resolution populations derived from those three lines, to fine-map the QTL affecting juvenile plant development as demonstrated in Schmalenbach et al. [19]. Additionally, there were several interesting QTL solely detected under low N treatment, for example, close to the dwarfing gene *arie*.*GP* on chromosome 5H. To conclude, our data indicate that the field performance of an adult barley genotype may be predictable by examining its juvenile development under hydroponic conditions. Thus, hydroponic studies, in particular to select genotypes tolerant to abiotic stresses, have a high potential to support modern plant breeding programs. In this regard, future fine mapping of the detected QTL in a hydroponic system, by means of phenotyping the already existing HR populations, which are derived from the original S42ILs, may be of great benefit both to breeders and plant physiologists in order to speed up selection of target phenotypes. The aforementioned three S42ILs on chromosome 7H may be excellent starting points for this endeavour.

## Abbreviations

ALL: Across treatments; HR: High-resolution; Hsp: *Hordeum vulgare* ssp. *spontaneum*; Hv: *Hordeum vulgare* ssp. *vulgare*; LSMeans: Least squares means; N: Nitrogen; N100: 100% N, i.e. control treatment; N25: 25% N, i.e. low N treatment; RP: Relative performance of an S42IL compared to 'Scarlett'; S42: BC_2_DH population derived from the cross 'Scarlett' x ISR42-8; S42IL: Introgression line derived from S42.

## Competing interests

The work was funded by the federal state of Saxony-Anhalt (FKZ: 3651A/0808, PI: Klaus Pillen).

## Authors' contributions

AH carried out the experiments in 2008, performed the joint data analysis and contributed substantially to writing the manuscript. AM carried out the experiments in 2011 and performed the data analysis in 2011. KP conceived the idea, supervised the project, contributed to data analysis and to writing the manuscript. All authors read and approved the final manuscript.
